# Bacterial Meningitis With Cerebral Edema in a Young Adult: A Simulation Case for Medical Students

**DOI:** 10.15766/mep_2374-8265.11354

**Published:** 2023-10-27

**Authors:** Kyle Cohen, Grant Gregory, James Nolin, Alexandra Sappington, Jonathan Hardy, Julia Alexander, Dianne Walker, John Giannini

**Affiliations:** 1 Third-Year Medical Student and Simulation Fellow, Alabama College of Osteopathic Medicine; 2 Instructor of Primary Clinical Skills, Alabama College of Osteopathic Medicine; 3 Assistant Professor of Radiology, Alabama College of Osteopathic Medicine; 4 Simulation Curriculum Coordinator, Alabama College of Osteopathic Medicine; 5 Associate Professor of Internal Medicine and Director of Simulation, Alabama College of Osteopathic Medicine

**Keywords:** Meningitis, Neuroradiology, *Streptococcus pneumoniae*, Critical Care Medicine, Emergency Medicine, Infectious Disease, Neurology, Simulation

## Abstract

**Introduction:**

Simulation in the preclinical medical education setting is a beneficial tool for students to develop clinical skills, supplement preexisting knowledge, and prepare for clinical rotations and beyond. We detail the complete simulation scenario, including a participant postresponse questionnaire, of a 28-year-old male who developed bacterial meningitis after experiencing an upper respiratory infection in the days prior.

**Methods:**

Simulation fellows and faculty at the Alabama College of Osteopathic Medicine created a simulation scenario pertaining to bacterial meningitis. The scenario utilized a high-fidelity patient simulator, one standardized participant for patient voiceover, one standardized participant as a patient family member, and one standardized participant as a physician consultant on an as-needed basis. Sixteen preclinical medical students from various specialty interest groups were recruited to participate in the scenario and complete the postscenario questionnaire.

**Results:**

The simulation scenario was well received by the participants, and 15 of 16 completed the postscenario questionnaire. Ninety-three percent strongly agreed the simulation was a valuable clinical experience. Additionally, 73% of participants strongly agreed that the simulation experience was realistic, 80% strongly agreed that it tested their clinical reasoning ability, and 53% strongly agreed it was appropriate for their level of clinical knowledge.

**Discussion:**

Medical simulation is a valuable educational tool tailored to maximize student learning and supplement the traditional didactic curriculum. The successful development and implementation of our meningitis simulation case further supports the continued use of medical simulation in the preclinical setting.

## Educational Objectives

By the end of this activity, learners will be able to:

1. Implement appropriate resuscitation measures for an acutely ill patient.
2. Obtain appropriate history and physical exam to arrive at a differential diagnosis of meningitis.
3. Analyze laboratory results to arrive at a differential diagnosis of meningitis.
4. Analyze imaging results to arrive at a differential diagnosis of meningitis.
5. Evaluate the need for empiric antibiotics in a potential neurologic infection.
6. Evaluate the need for consultation with neurology and/or infectious disease.
7. Perform the procedural skill of lumbar puncture.
8. Perform the procedural skill of venous access.
9. Perform the procedural skill of endotracheal intubation.

## Introduction

Medical simulation has long been used as a tool in the preclinical years of medical school education as well as at higher levels of medical education. Simulation has been shown to be a valuable tool for teaching clinical reasoning to students prior to their reaching their clinical education experience.^1^ Utilizing a hybrid method of integrating clinical skills on low-fidelity task trainers in the setting of a high-fidelity clinical scenario has been shown to increase student confidence in performing those skills.^2^ Additionally, the use of high-fidelity simulation has been shown to increase student confidence while decreasing anxiety surrounding patient encounters.^3^

Meningitis is an inflammation of the meningeal layers surrounding the brain and spinal cord that has significant morbidity and mortality across all age groups.^4^ Based on our search, there has been one high-fidelity simulation case of pediatric meningitis^5^ published in *MedEdPORTAL* as well as several other resources, including videos^6,7^ and problem-based learning activities.^8,9^ However, there have been no simulated cases of an adult patient with meningitis published in *MedEdPORTAL*. We believe that performing this case as a high-fidelity simulation experience enhances the realness of the case and brings about a strong sense of teamwork and urgency to manage the acutely ill patient, which may not be obtained by watching a video or participating in a problem-based learning activity.

This case was developed for first- and second-year medical students as an extracurricular event at the Alabama College of Osteopathic Medicine, but the scenario could easily translate into an effective learning modality for other curricula and be integrated into an existing medical simulation curriculum. The scenario can be utilized by medical schools, physician assistant schools, nurse practitioner programs, and medical residency programs as an effective resource to train the next generation of health care providers in achieving proficiency in the management of bacterial meningitis.

This case presents a few elements not yet experienced by first- and second-year medical students. First is the idea of an uncommon presentation of a common disease. It is well known that many common diseases have a typical presentation, or a triad, pentad, and so on. However, it is also well known that many of these classic presentations are never fully fulfilled and that a patient may exhibit only one or two of the classic findings. This case is a prolonged meningitis case with concomitant cerebral edema, and the presenting condition can obscure the classical findings of meningitis. It is important for medical students to begin experiencing cases that are outside the classic presentation early in their education to help them develop more robust differential diagnoses as they progress. Second, medical students are accustomed to obtaining history directly from the patient. In this case, the patient is unresponsive, and the history must be obtained from a secondary source, the spouse.

## Methods

The simulation scenario, including the generated debrief material, is outlined in its entirety in Appendices A–E. The case was completed as an extracurricular event and had some time constraints. While what follows is the format we utilized, the format and flow may be adjusted based on the needs of different participants.

### Participants

This extracurricular simulation event included eight first-year and eight second-year medical students and took place in the spring of 2022. No explicit prereading was required for these participants as they had all successfully completed the neurology course of the medical school curriculum, which addressed neurologic infectious diseases (meningitis) and included diagnosis, management, and possible complications.

### Development

The goal was to develop a meningitis scenario that was challenging for first- and second-year medical students yet still provided sufficient information for them to arrive at the appropriate differential diagnosis and treatment. The scenario was developed based on the case report of Pinilla-Monsalve and colleagues involving atypical streptococcal meningitis that ultimately led to meningitis with concomitant cerebral edema.^10^ While the original case report resulted in the demise of the patient, our goal was to allow for improvement if the appropriate management was administered in a timely manner. Therefore, the facilitators of the case needed a working knowledge of pathophysiology and management of both meningitis and increased intracranial pressure due to cerebral edema. The flow and description of the simulated case, as well as the patient's history, are available in the simulation case and facilitator guide (Appendix A).

### Equipment/Environment

This scenario was developed using a high-fidelity patient simulator, and the coinciding software was programmed using the case flow sheet included in Appendix A. During the scenario, the participants were offered the contents of a crash cart, including airway management devices, oxygen delivery devices, and a simulated defibrillator. Medication administration was verbalized by the learners but could easily be adapted to give them the chance to administer the medications themselves. At the beginning of the encounter, participants were provided with the brief triage information (included in Appendix A) displayed on the patient monitor in the room. The patient simulator was lying supine in a hospital bed wearing a baseball cap. Throughout the encounter, participants could order imaging studies with or without imaging reports (Appendix B) and various labs (Appendix C). The imaging reports were provided only if the participants requested a read of the image or a consultation with radiology. The 12-lead EKG used was the standard EKG within the simulation software. The learners also conducted the tasks of lumbar puncture (LP), intravenous access (IV), and endotracheal intubation (ET) on low-fidelity task trainers during sessions separate from the simulated patient encounters. A more detailed list of equipment is available in the facilitator guide (Appendix A).

### Personnel

During our event, we ran two simulations simultaneously, thus requiring double the personnel. The description of the following personnel is for one simulation. The simulated patient encounter utilized one facilitator and one standardized participant during the encounter. The facilitator operated the simulated patient and patient monitor while the standardized participant acted as the patient's spouse, who was available by phone. Both the facilitator and the standardized participant observed the encounter from the simulation control room. The standardized participant acting as the spouse provided history to the participants using the speaker system in the patient room. At the conclusion of the scenarios, the facilitators conducted the debrief.

### Implementation

A 5-minute prebrief session was held to inform the participants of the flow of the event. Participants randomly assigned themselves to groups of four, were given a letter assignment (A-D), and identified group members with the following roles: team captain, history taker, physical examiner, and scribe. Participants were briefed that the team captain was the only team member allowed to request labs, imaging, interventions (IVs, fluids, and medications), and any consults. However, the teams were encouraged to discuss these orders prior to the captain administering a request. This emphasized the need for teamwork and effective communication. In the interest of time, the participants were briefed to verbalize any advanced airway procedures but could perform basic tasks such as administering oxygen and bag mask ventilation themselves.

Two scenarios were run simultaneously using separate simulation rooms, facilitators, and standardized patients. To stay within our time constraints, the other two teams worked with additional faculty on skills practice during that same 20 minutes. At the conclusion of the 20 minutes, the teams rotated so that those who completed the scenario first went to skills practice and vice versa. At the conclusion of the second 20-minute period, there was a 20-minute debrief. The Figure contains a detailed schedule of the event as it was run.

**Figure.**
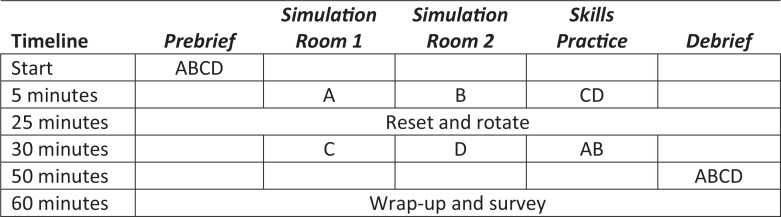
Detailed schedule of the simulation event. The letters A, B, C, and D denote the groups of learners and the appropriate rotation.

When the learners entered the room, they were given the background information included in the case flow sheet in Appendix A. Throughout the encounter, facilitators utilized the case flow sheet to keep track of learners ordering labs (Appendix C), medications, and imaging (Appendix B). As learners ordered these items, they were either presented on the patient monitor or verbalized by the facilitator in a timely manner 1–2 minutes after the order had been placed. There was no preset function of the simulation other than the deterioration of the patient at the 8-minute mark if no appropriate intervention had been given up to that point. All other progressions of the scenario were based on the orders placed by the learners. If they consulted any specialty, the facilitator operating the simulator and monitor acted as the consult on the phone and provided any needed feedback or was able to take the patient report as part of the final disposition.

The event also involved practicing LP, IV, and ET skills on respective low-fidelity task trainers. The skills practice session was a hands-on opportunity for learners to practice these skills with direct feedback from faculty and facilitators. While this extra skills practice was not a necessary component of the simulation event, it served as an added opportunity to allow participants to practice these hands-on skills early in their education as well as filling in what otherwise would have been idle time for the two groups not actively participating in the simulated patient encounter. The first-year learners had previously been trained on LP, while the second-year learners had been trained on all three skills as part of the medical school curriculum. Facilitators and faculty members were available to assist with these skills and utilized the “see one, do one” technique.

### Debriefing

To debrief the session following completion of the scenario, we employed the plus/delta model,^11^ which began by asking students what had gone well and what they felt they needed to improve on. Specific debriefing questions are included in Appendix E. Following a general overview of the scenario, a PowerPoint presentation was utilized to highlight key points of the encounter (Appendix E).

### Assessment

Critical actions were identified as follows:

• Check airway, breathing, and circulation.
• Obtain and interpret vital signs.
• Initiate primary resuscitation by obtaining/requesting IV access and giving fluid bolus.
• Administer antipyretic.
• Obtain pertinent history from spouse.
• Obtain labs (complete blood count, basic metabolic panel, liver function, coagulation profile, urinalysis).
• Obtain blood cultures.
• Perform LP.
• Request head CT.
• Consider ET.
• Request postintubation chest X-ray if ET done.
• Administer broad spectrum antibiotics (not required to know specific drug).
• Consult neurology/infectious disease for the management of meningitis.
• Admit to intensive care unit.
• Work effectively as a team.

These critical actions were not explicitly evaluated during our simulated encounter but were discussed as part of the debriefing process. However, the checklist can be used to evaluate the learners during the encounter and is included in the facilitator guide (Appendix A). At the conclusion of the event, the learners provided feedback on a voluntary basis using a postencounter questionnaire. Many of these questions were scored using a 5-point Likert scale (1 = *strongly disagree,* 5 = *strongly agree*), and the learners had the chance to evaluate the case to ensure the objectives had been met. We updated the postencounter questionnaire (Appendix D) after the implementation; it now contains questions not deployed with the learners described here. The questionnaire was adapted using the Simulation Effectiveness Tool—Modified developed by Leighton and colleagues.^12^

## Results

Before the scenario was completed by preclinical medical students, faculty and staff piloted the case to ensure quality control. No pilot errors were identified in the testing phase. Afterwards, the scenario was completed by four teams of learners who also filled out the postscenario questionnaire following the conclusion of the training. A detailed breakdown of the questionnaire results is fetaured in the Table.

**Table.**
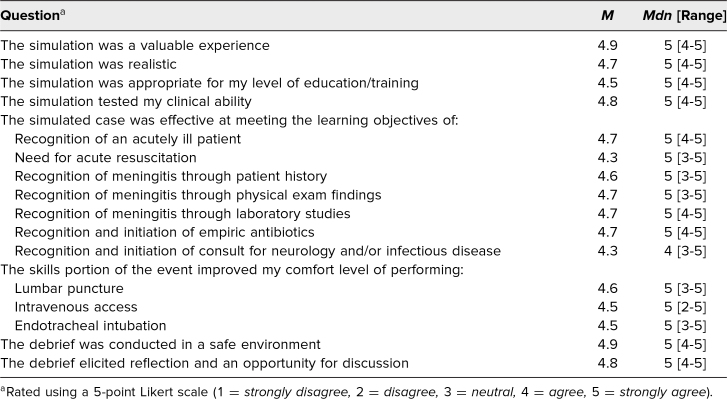
Simulation Evaluation Results (*N* = 15)

Fifteen of the 16 learners completed the postscenario questionnaire. Participants were a mix of first- and second-year medical students, and it is worth noting that their respective responses to the survey were slightly different on several questions. Overall, the scenario was positively received, with 93% strongly agreeing that the simulation was a valuable experience. Similarly, 73% strongly agreed that the simulation was realistic, and one learner commented, “I will feel more comfortable when I experience this out in the real world.” Eighty percent strongly agreed the simulation tested their clinical reasoning ability. Of note, only 53% strongly agreed the simulation was appropriate for their level of education (43% of first-year and 63% of second-year learners). It is possible that in our small sample, the first-year students felt less comfortable with the critical care aspect of the case.

Relating to the questions regarding the learning objectives, 73% of respondents strongly agreed that the simulation helped them recognize an acutely ill patient while only 53% strongly agreed it helped determine the need for acute resuscitation. The distribution was equal for the responses to the recognition of meningitis through patient history (*M* = 4.6), physical exam findings (*M* = 4.7), and laboratory findings (*M* = 4.7), as well as the recognition and initiation of empiric antibiotics (*M* = 4.7). One learner commented, “It made me self-aware of how I need to focus on a thorough history and become more proficient at interpreting lab values in critical situations,” while another mentioned that the simulation helped them to “better recognize the signs and symptoms of meningitis, as well as when to intubate a patient.” However, only 33% strongly agreed the simulation was effective at recognition and initiation of a consult with neurology or infectious disease. While there were several neutral responses throughout, there were no disagree or strongly disagree responses to these questions.

The practice session provided similar results in overall improved comfort level with performing the skills. For the LP, 67% of learners strongly agreed with improved comfort level; however, there was a discrepancy between average rating for first-year learners (*M* = 4.3) and second-year learners (*M* = 4.9). Similarly, 60% of learners strongly agreed that the ET station improved their comfort level, again with a discrepancy between first-year learners (*M* = 4.3) and second-year learners (*M* = 4.6). For the IV station, 73% strongly agreed with improved comfort level, but there was a swap in the discrepancy seen in the two previous skills, with first-year learners (*M* = 4.7) reporting more improved comfort than second-year learners (*M* = 4.4).

The debrief session was also evaluated on the questionnaire. The overwhelming majority of respondents (87%) strongly agreed that the debrief was conducted in a safe environment. Similarly, 80% strongly agreed that the debrief elicited appropriate reflection and an opportunity for discussion.

## Discussion

This scenario was developed to train preclinical medical students on the uncommon etiology and classical manifestations of severe bacterial meningitis with concomitant increased intracranial pressure. Although teams performed at various levels during the simulation encounter, we feel that all students left the training scenario with a better understanding of bacterial meningitis, including how to recognize, diagnose, and manage its potential complications.

Reflecting on the training scenario, it is possible that recency bias may have been a driving factor for some of the outcomes observed. Teams were composed of a mix of first- and second-year students. At our institution, the neurology course, taught during the spring semester of the first year, had wrapped up months prior to the first-year participants completing our training scenario. As the survey questions reflected the abilities of the case rather than the abilities of the learners, there were no data to reflect individual learner capabilities; instead, learners simply reported whether the case had addressed the critical actions. Anecdotally, we found that second-year students noticed and appropriately responded to critical events such as crashing vital signs, while first-year students tended to understand pathophysiology, etiologic origins, and the recommended empirical treatment of bacterial meningitis. We believe it is beneficial for all students to familiarize themselves with working with different graduating classes during simulation scenarios, as clinical rotations and residency will comprise health care teams of differing experience levels.

Based on the results, we feel the event was an overall success. However, we did notice a trend within the small sample when the first-year medical students reported the case was appropriate for their learning level less frequently than did their second-year counterparts. It is unclear, however, which aspects of the case were not appropriate. This may reflect the progression through education, as the first-year learners had completed only the neurology course and not the cardiac or pulmonary courses. The second-year learners may have had a stronger basis for the initial resuscitation and overall management of the case. Therefore, the case may be better suited for learners who are toward the end of their initial education, such as second-year medical students, final-semester nurse practitioner students, and second-year physician assistant students. There is certainly an opportunity for further investigation using a questionnaire addressing areas of the case that may be more difficult for, or outside the scope of, participants.

Additionally, we noted discrepancies in the distribution of scores related to the skills stations. These may have been due to recency bias as well. For the LP and ET stations, the second-year learners appeared to receive more benefit than the first-year learners, which may have been multifactorial. Related to the LP, the first-year learners had recently completed the neurology course and, due to their fresh learning of the skill, may not have perceived its practice to be as useful an experience. The second-year learners had just completed the pulmonary course and initial training on the ET skills, yet they reported an increased level of comfort as a result of the event. Conversely, the first-year learners reported increased comfort relating to the IV skill station, though they had not been formally trained as part of their curriculum. It is possible that there was a dilution of the experience due to the time constraints we had for the event. Whether added time would have affected these results is unknown and may be a consideration for future implementations of this simulation.

Following the event, we noticed that the skills portion may have been a limiting factor. If the event were run again, one recommendation would be to modify the skills portion or remove it completely. A possible revision would be having all participants complete the skills workshop portion prior to the simulated case. This would allow more time for the participants to learn and practice these skills. Subsequently, the skills could be implemented directly in the simulated case. However, due to our time constraints, this was not a possibility for us. Another option for the skills portion, if equipment and faculty allow, would be to have more resources available for participants. We had limited faculty available for the extracurricular event and utilized one faculty for each of the three skills stations as participants rotated through. There is potential to expand this to have multiple skills stations.

The aims of our simulation event were to determine if a simulated patient encounter around an atypical presentation of bacterial meningitis could allow a student to achieve the desired learning objectives and to provide an adult meningitis simulation case to the education community. We felt it was important for participants to evaluate the case's effectiveness in a subjective manner to ensure that it met their expected needs prior to implementing it as part of a graded or faculty-evaluated event. We did not use it as part of our curriculum and therefore did not evaluate the learners’ abilities during the simulated patient encounter. However, it may be beneficial to conduct further research into using this case as a learning tool and utilizing the simulation checklist in Appendix A as well as the learning objectives as components for objective measurement by observing faculty or facilitators. Additionally, it may be prudent, when evaluating the case in subjective measures in the future, to allow participants to rate their comfort level or increased knowledge, as well as to elaborate on these areas with opportunity for comment.

Another potential limitation we noticed was that the survey did not ask which activities were most and least beneficial. Having participants rate the activities from most to least useful could drive future events and allow faculty to develop them around activities that students value. We also recommend questioning participants regarding whether the allotted time is sufficient for the training. Additionally, a free-text option could be added for participants to comment on areas of the event they would like to see changed if they were to participate in subsequent similar events. A suggested postencounter questionnaire has been included in Appendix D, as an improvement based on these reflections.

We feel this simulated patient encounter and/or event could be easily adapted to fit the needs of other programs. For instance, we ran two simulated encounters simultaneously, as we felt we had adequate staffing and were under time constraints. It is possible to run a single scenario if time allows and there are limited faculty. Conversely, if adequate resources are available, the scenario could be run simultaneously as many times as needed. While we utilized a separate skills practice session as a time filler for the alternating groups and did not implement the skills within the scenario due to time constraints, it is possible to implement these skills in the scenario to replicate an entire beginning-to-end encounter rather than verbalizing intervention. After holding this event, our recommendations for future use are solely dependent on the availability of resources and faculty/facilitators as well as on the time constraints of an individual program. It is our hope that this simulated patient encounter offers an opportunity for others to replicate or adapt the information provided for the specific needs of their programs, either as an extracurricular event or implemented within a curriculum with objective measures.

## Appendices


Simulation Case and Facilitator Guide.docxSimulation Images.docxLaboratory Values.docxPostencounter Questionnaire.docxMeningitis Debrief.pptx
*All appendices are peer reviewed as integral parts of the Original Publication.*
