# Supplementary material for: Bacterial Meningitis With Cerebral Edema in a Young Adult: A Simulation Case for Medical Students
Source: MedEdPORTAL. 2023 Oct 27;19:11354. doi: 10.15766/mep_2374-8265.11354 (PMC10603216; doi:10.15766/mep_2374-8265.11354)
Supplement: Supplementary file 1 — Simulation Case and Facilitator Guide.docxSimulation Images.docxLaboratory Values.docxPostencounter Questionnaire.docxMeningitis Debrief.pptx [file mep_2374-8265.11354-s001.zip › B. Simulation Images.docx]

Appendix B: Simulation Images

Pre-intubation CXR


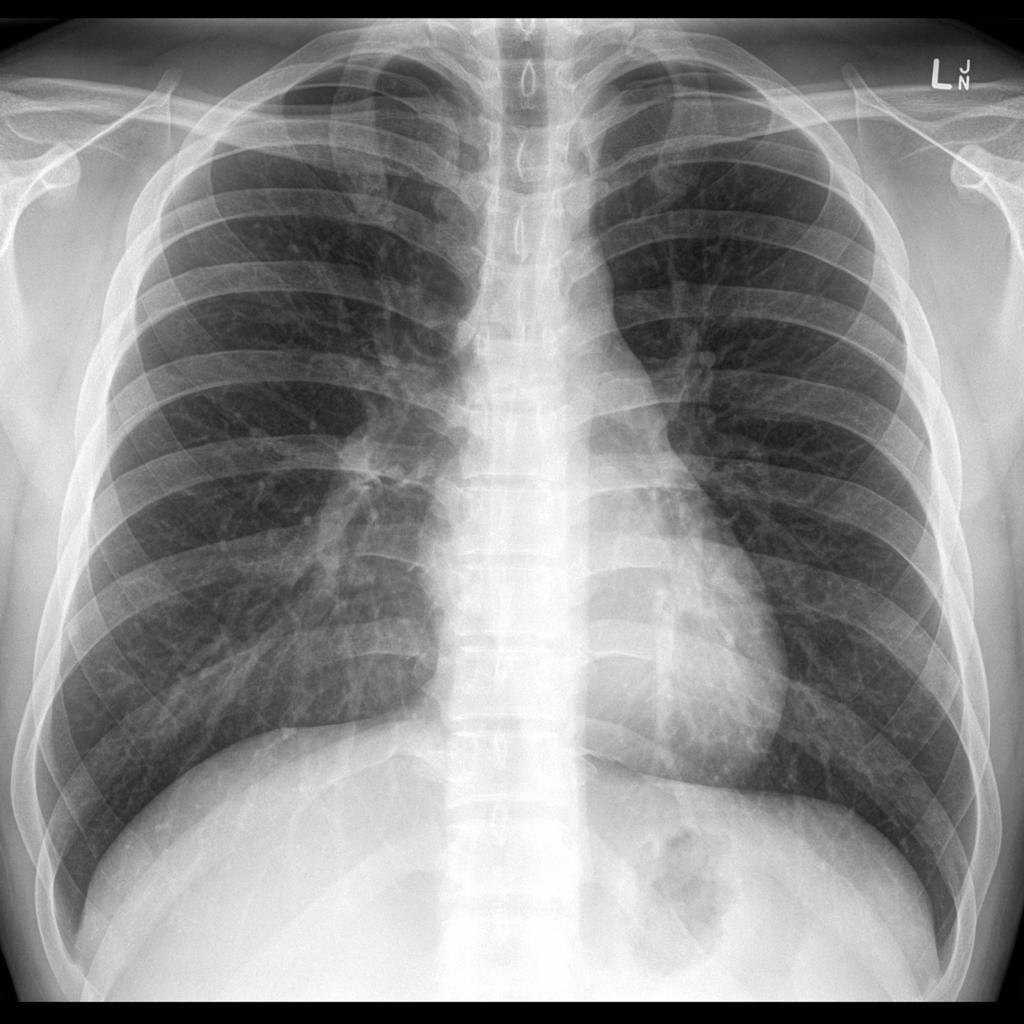


Image by Gaillard, F., retrieved from <https://radiopaedia.org/cases/normal-frontal-chest-x-ray> on 19 Aug 2021. Creative Commons License Associated: CC BY-NC-SA 3.0

Post-intubation CXR


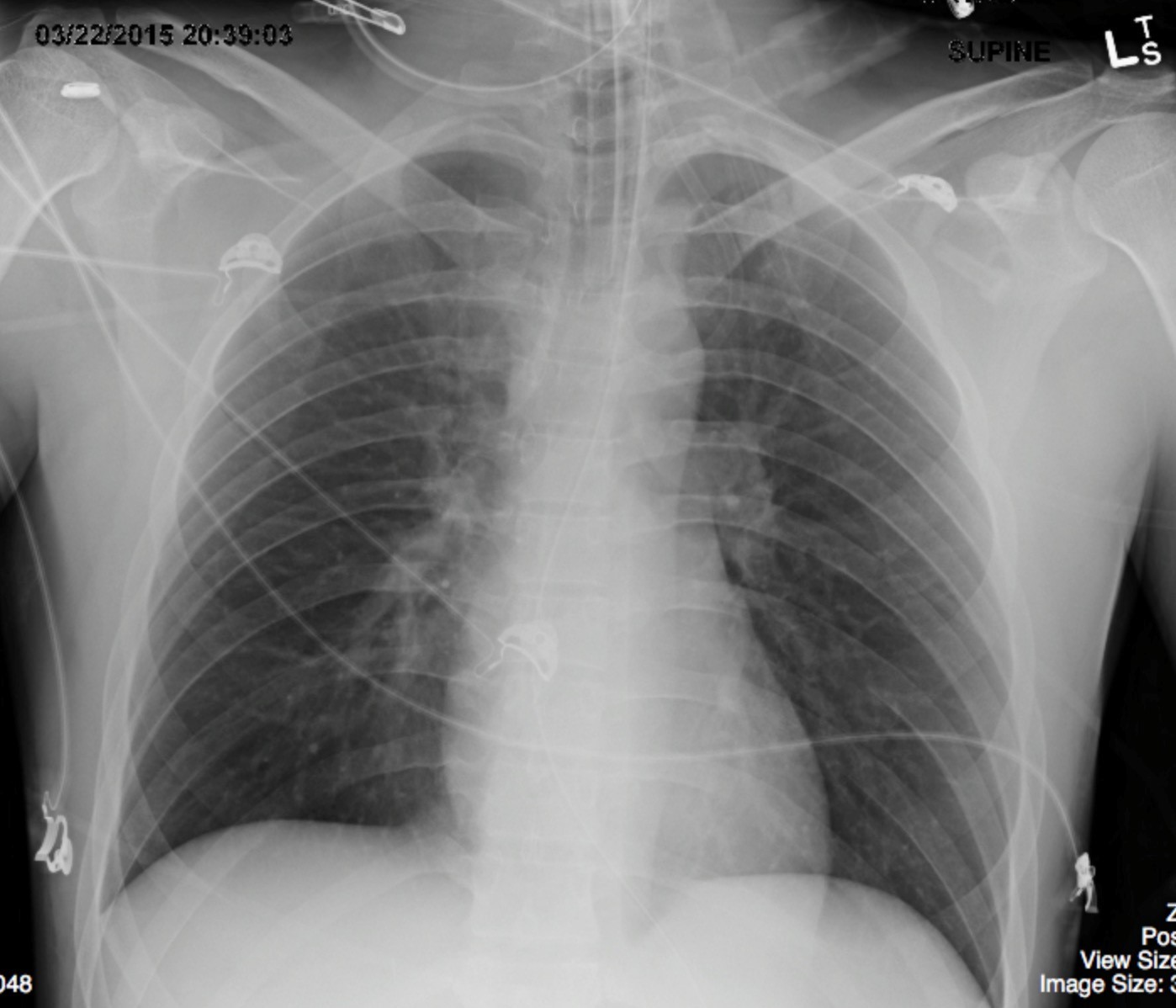


Image by Burbridge, B., retrieved from <https://undergradimaging.pressbooks.com/chapter/tubes-and-catheters/> on 19 Aug 2021. Creative Commons License associated: CC BY-NC-SA 4.0

Head CT


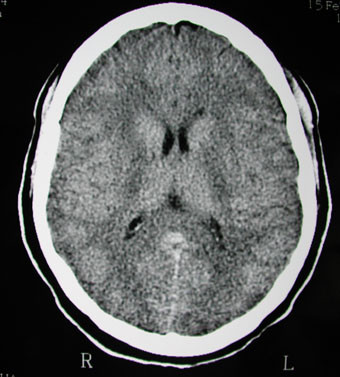


Image by Lefebvre N, Forestier E, Farhi D, Mohseni-Zadeh M, Remy V, Lesens O, Christmann D, & Hansmann Y retrieved from <https://jmedicalcasereports.biomedcentral.com/track/pdf/10.1186/1752-1947-1-22.pdf> on 19 Aug 2021. Creative Commons License Associated: CC BY 2.0

Head CT Interpretation (Hints toward meningitis)

Technique: Multiple axial 5mm noncontrast images of the head

Findings:

There is no hydrocephalus. There is diffuse loss of gray-white differentiation throughout the brain with cortical effacement. There is near complete obliteration of the basal cisterns, in keeping with increased intracranial pressure. There are no areas of hemorrhage. There are no extra-axial fluid collections.

Impression: Findings in keeping with diffuse increase in intracranial pressure as detailed above. Correlate clinically to rule out meningitis or other causes of cerebral edema.

Head CT Interpretation (Does not hint toward meningitis)

Technique: Multiple axial 5mm noncontrast images of the head

Findings:

There is no hydrocephalus. There is diffuse loss of gray-white differentiation throughout the brain with cortical effacement. There is near complete obliteration of the basal cisterns, in keeping with increased intracranial pressure. There are no areas of hemorrhage. There are no extra-axial fluid collections.

Impression: Findings in keeping with diffuse increase in intracranial pressure as detailed above.
