# Supplementary material for: Bacterial Meningitis With Cerebral Edema in a Young Adult: A Simulation Case for Medical Students
Source: MedEdPORTAL. 2023 Oct 27;19:11354. doi: 10.15766/mep_2374-8265.11354 (PMC10603216; doi:10.15766/mep_2374-8265.11354)
Supplement: Supplementary file 1 — Simulation Case and Facilitator Guide.docxSimulation Images.docxLaboratory Values.docxPostencounter Questionnaire.docxMeningitis Debrief.pptx [file mep_2374-8265.11354-s001.zip › C. Laboratory Values.docx]

Appendix C. Laboratory Values

Patient: Nicholas Payne

| BASIC METABOLIC PANEL | | REFERENCE RANGE |
| --- | --- | --- |
| Sodium | 136 | 135-147 mmol/L |
| Potassium | 5 | 3.5-5.2 mmol/L |
| Chloride | 100 | 95-107 mmol/L |
| CO2 | 25 | 22-30 mmol/L |
| Urea Nitrogen (BUN) | 50 | 7-20 mg/dL |
| Creatinine | 2.5 | 0.5-1.2 mg/dL |
| Glucose | 100 | 60-110 mg/dL |

| COMPLETE BLOOD COUNT | | REFERENCE RANGE |
| --- | --- | --- |
|  |  | MALE FEMALE |
| White Blood Cell (WBC) | 15,000 | 4,500 – 10,000 K/uL |
| Hemoglobin (HGB) | 12.8 | 13.5 – 16.5 g/dL 12.0-15.0 g/dL |
| Hematocrit (HCT) | 35 | 41 – 50% 36 – 44% |
| Platelet | 120 | 100,000 – 450,000 K/uL |

| LIVER FUNCTION | | REFERENCE RANGE |
| --- | --- | --- |
| Albumin | 40 | 35 – 50 g/L |
| Alk Phos | 85 | 50 – 100 U/L |
| ALT | 35 | 5 – 30 U/L |
| AST | 30 | 5 – 30 U/L |
| Total Bilirubin | 15 | 2 – 20 mmol/L |

| COAGULATION PROFILE | | REFERENCE RANGE |
| --- | --- | --- |
| Partial thromboplastin time (PTT) | 45 | 30 – 45 sec |
| Prothrombin time (PT) | 12 | 10 – 12 sec |
| International Normalized Ratio (INR) | 2 | 1 - 2 |

| Urinalysis | | REFERENCE RANGE |
| --- | --- | --- |
| Color | Dark yellow | Pale, dark yellow, amber |
| Ph | 7 | 5-8 |
| Specific gravity | 1.030 | 1.002-1.035 |
| Protein | Trace | Negative/Trace |
| Glucose | Negative | Negative |
| Blood | Negative | Negative |
| Nitrites | Negative | Negative |
| Leukocyte | Negative | Negative |
| Microscopic | 0 | 0-2/HPF |

Patient: Nicholas Payne

| Lumbar Puncture | | REFERENCE RANGE |
| --- | --- | --- |
| Color | Cloudy | Clear |
| RBCs | 9000 | 0 |
| WBCs | 362 | 0-5 cells/μL |
| Neutrophils | 85% | <2% |
| Protein | 137 | <45 mg/dL |
| Glucose | 40 | >60% of serum glucose |
| CSF Lactate | 3.9 | 1.1-2.4 mmol/L |
| Pressure | 15 | 10-15 cm H_2_O |

These labs are not shown to students

| Toxicology Screen | | REFERENCE RANGE |
| --- | --- | --- |
| Acetaminophen | <10 | <10 |
| Salicylates | <5 | <5 |
| EtOH | 0 | 0 |
| Opiates | neg | negative |
| Cocaine Metabolites | neg | negative |
| Benzodiazepines | neg | negative |
| Cannabis | neg | negative |
| Barbiturates | neg | negative |
|  |  |  |

| Verbal Lab Results | | REFERENCE RANGE |
| --- | --- | --- |
| C-Reactive Protein | 300 | 0.8 – 3.0 mg/L |
| Serum Lactate | 3 | 0.5 – 2.2 mmol/L |
