# Supplementary material for: Bacterial Meningitis With Cerebral Edema in a Young Adult: A Simulation Case for Medical Students
Source: MedEdPORTAL. 2023 Oct 27;19:11354. doi: 10.15766/mep_2374-8265.11354 (PMC10603216; doi:10.15766/mep_2374-8265.11354)
Supplement: Supplementary file 1 — Simulation Case and Facilitator Guide.docxSimulation Images.docxLaboratory Values.docxPostencounter Questionnaire.docxMeningitis Debrief.pptx [file mep_2374-8265.11354-s001.zip › E. Meningitis Debrief.pptx]

## Slide 1
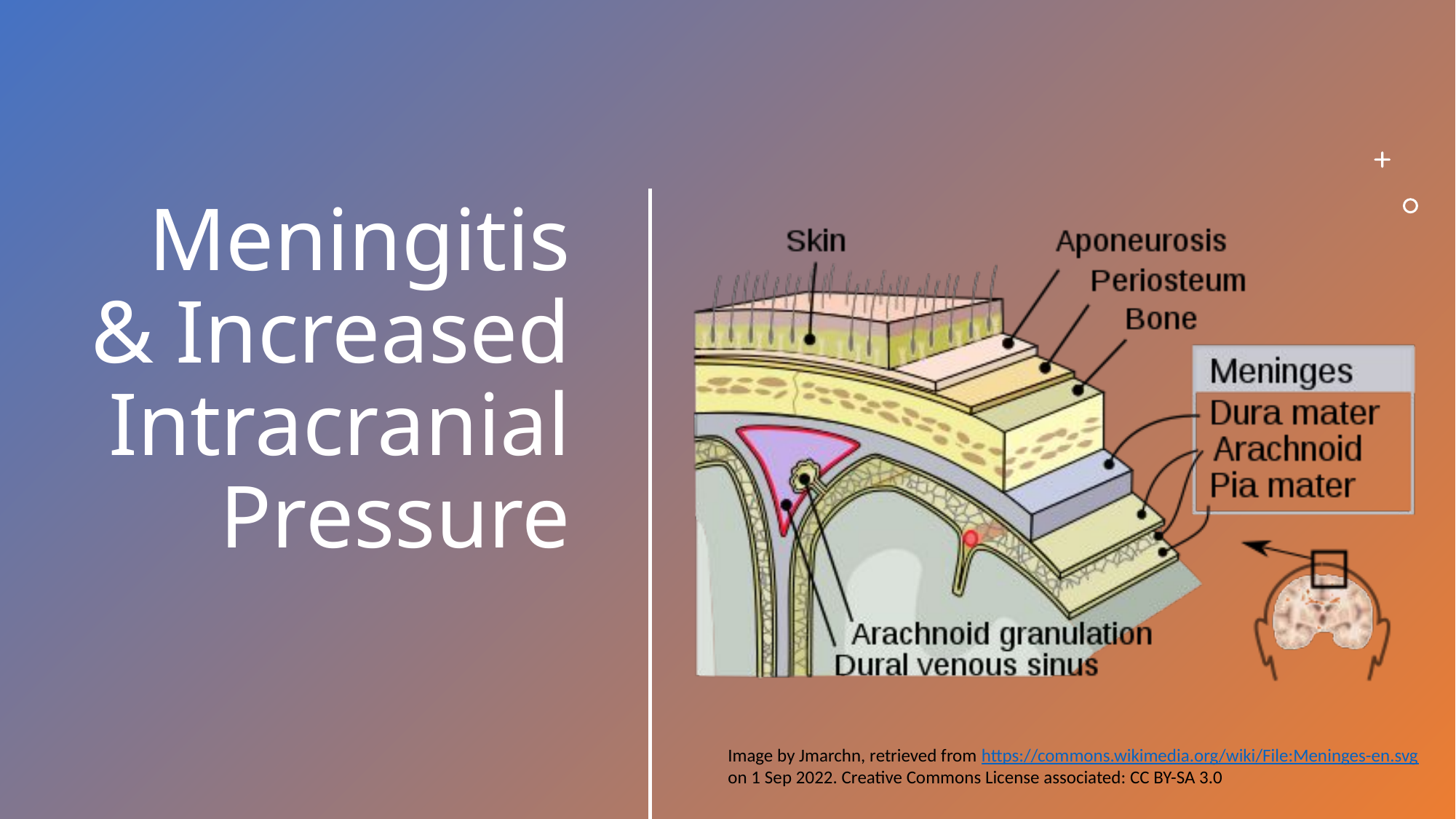

# Meningitis & Increased Intracranial Pressure
Image by Jmarchn, retrieved from https://commons.wikimedia.org/wiki/File:Meninges-en.svg on 1 Sep 2022. Creative Commons License associated: CC BY-SA 3.0

## Slide 2
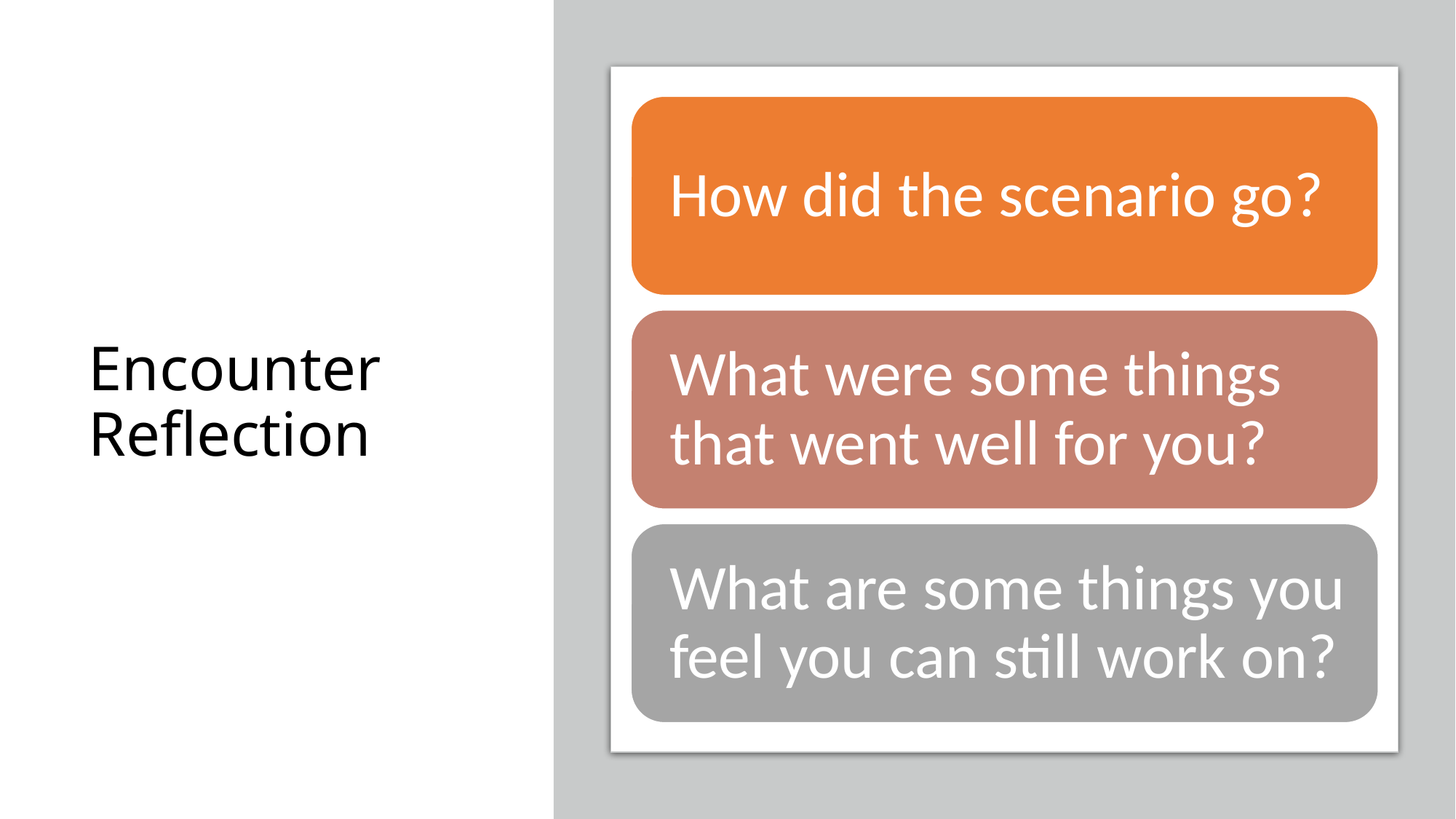

# Encounter Reflection

## Slide 3
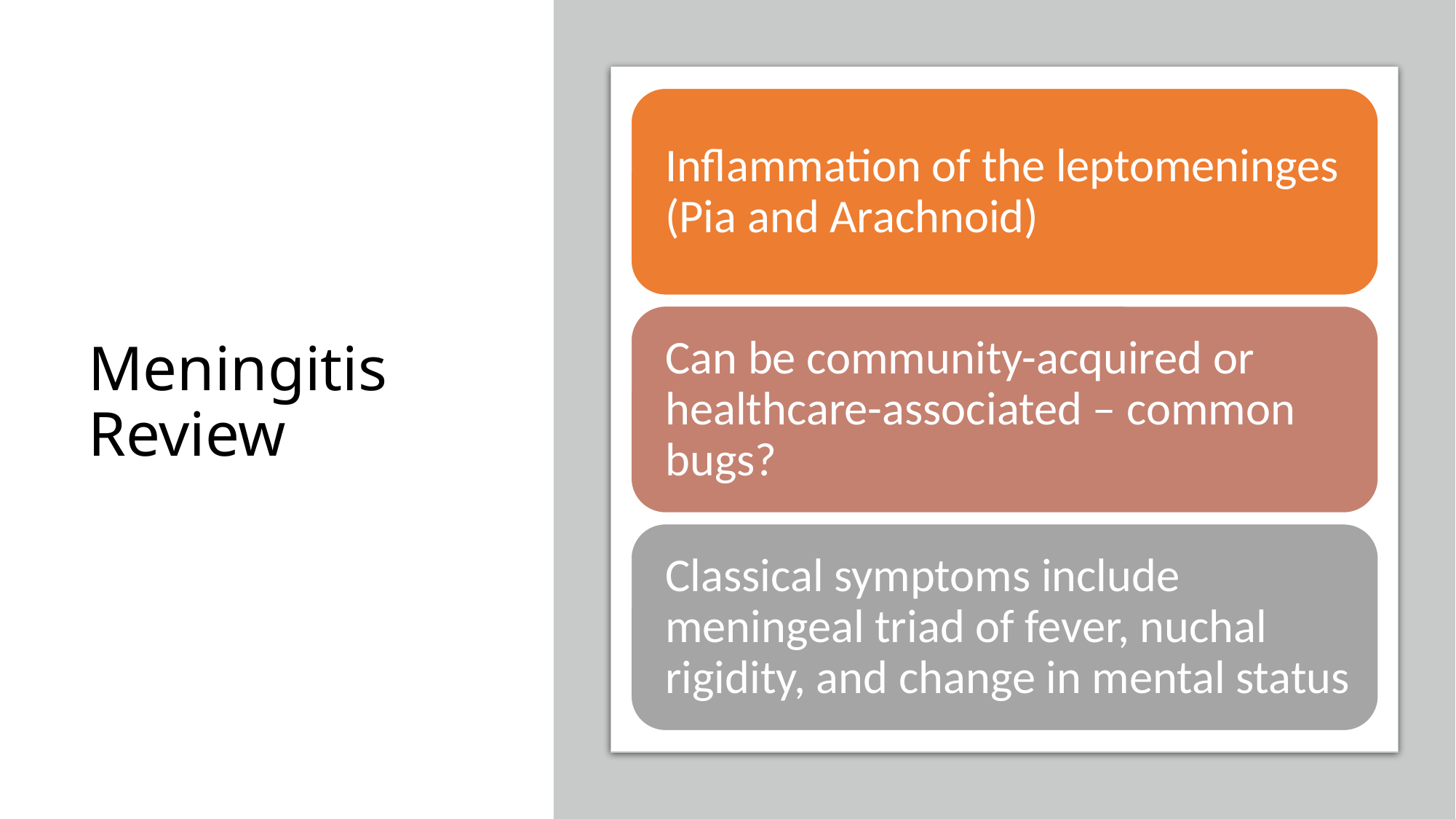

# Meningitis Review

## Slide 4
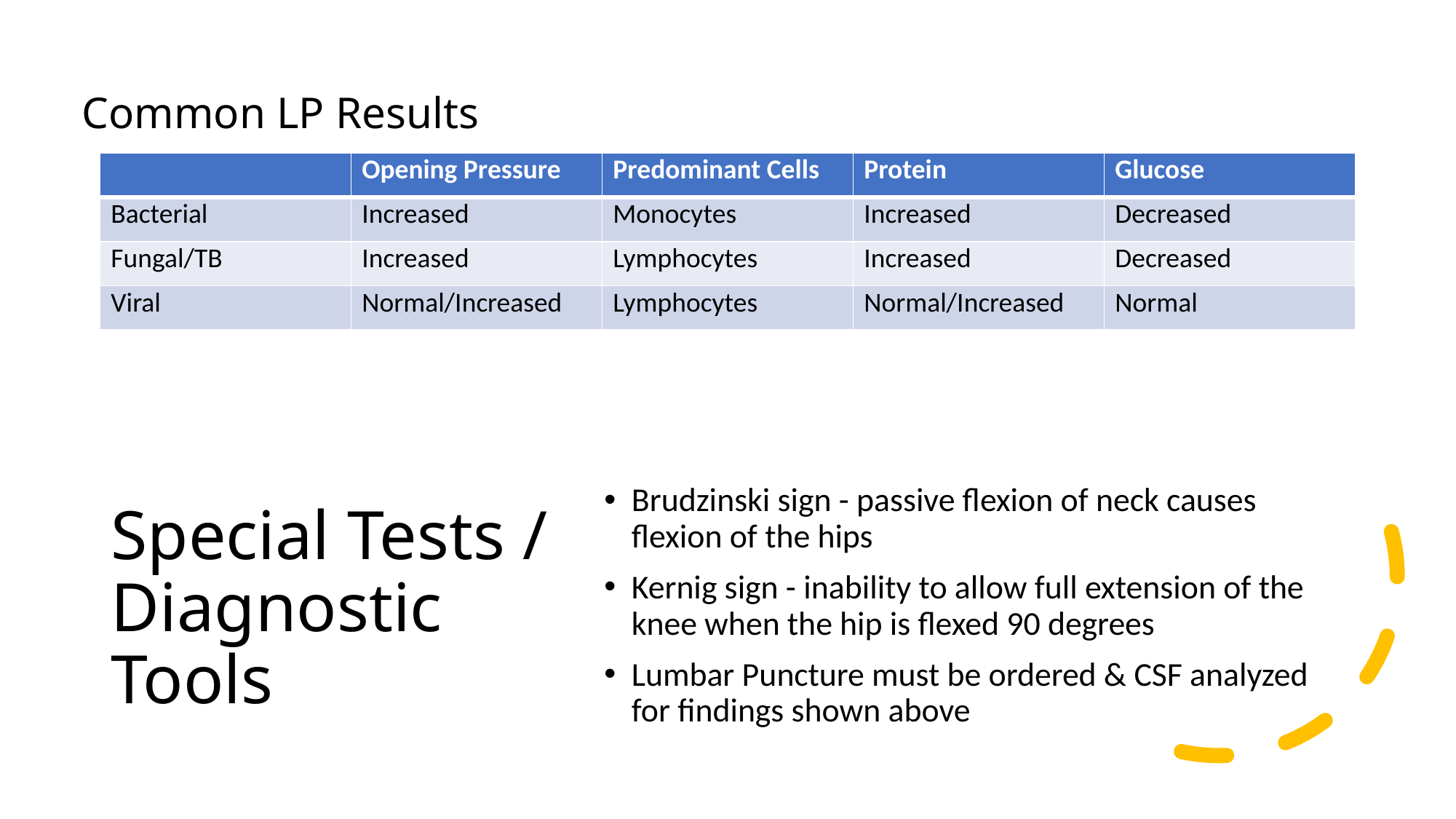

Common LP Results
| | Opening Pressure | Predominant Cells | Protein | Glucose |
| --- | --- | --- | --- | --- |
| Bacterial | Increased | Monocytes | Increased | Decreased |
| Fungal/TB | Increased | Lymphocytes | Increased | Decreased |
| Viral | Normal/Increased | Lymphocytes | Normal/Increased | Normal |
# Special Tests / Diagnostic Tools
Brudzinski sign - passive flexion of neck causes flexion of the hips
Kernig sign - inability to allow full extension of the knee when the hip is flexed 90 degrees
Lumbar Puncture must be ordered & CSF analyzed for findings shown above

## Slide 5
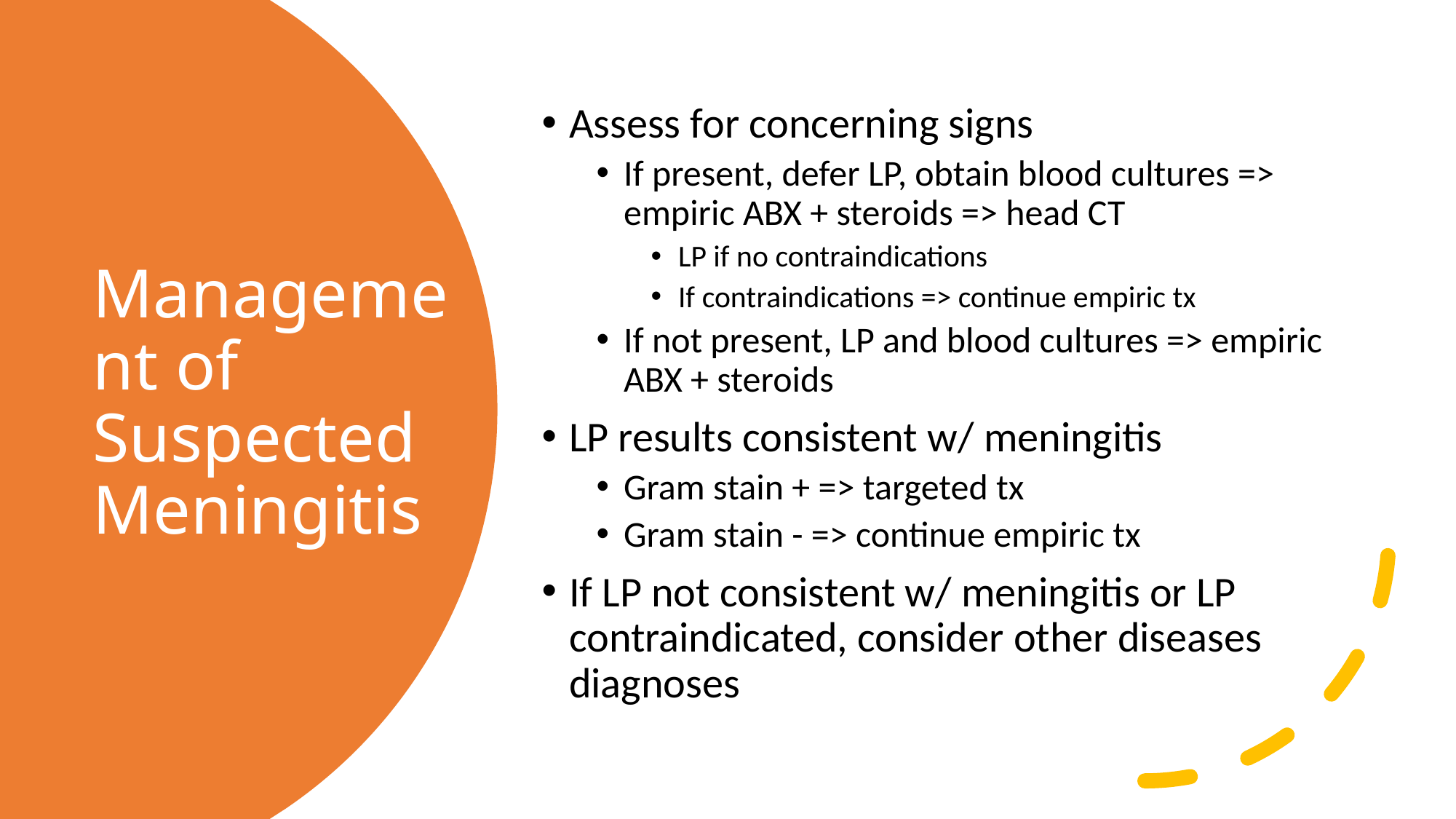

Assess for concerning signs
If present, defer LP, obtain blood cultures => empiric ABX + steroids => head CT
LP if no contraindications
If contraindications => continue empiric tx
If not present, LP and blood cultures => empiric ABX + steroids
LP results consistent w/ meningitis
Gram stain + => targeted tx
Gram stain - => continue empiric tx
If LP not consistent w/ meningitis or LP contraindicated, consider other diseases diagnoses
# Management of Suspected Meningitis

## Slide 6
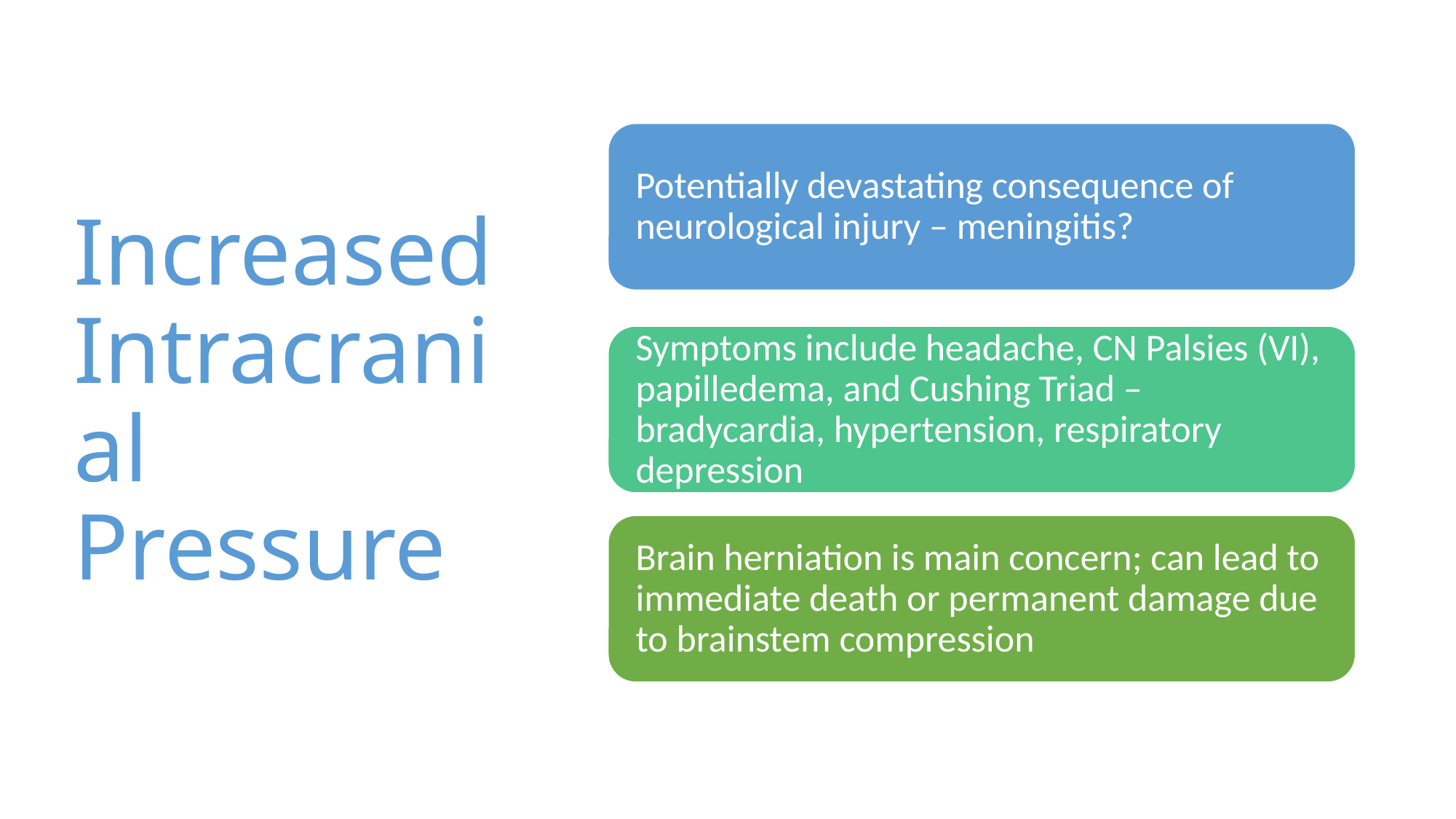

# Increased Intracranial Pressure

## Slide 7
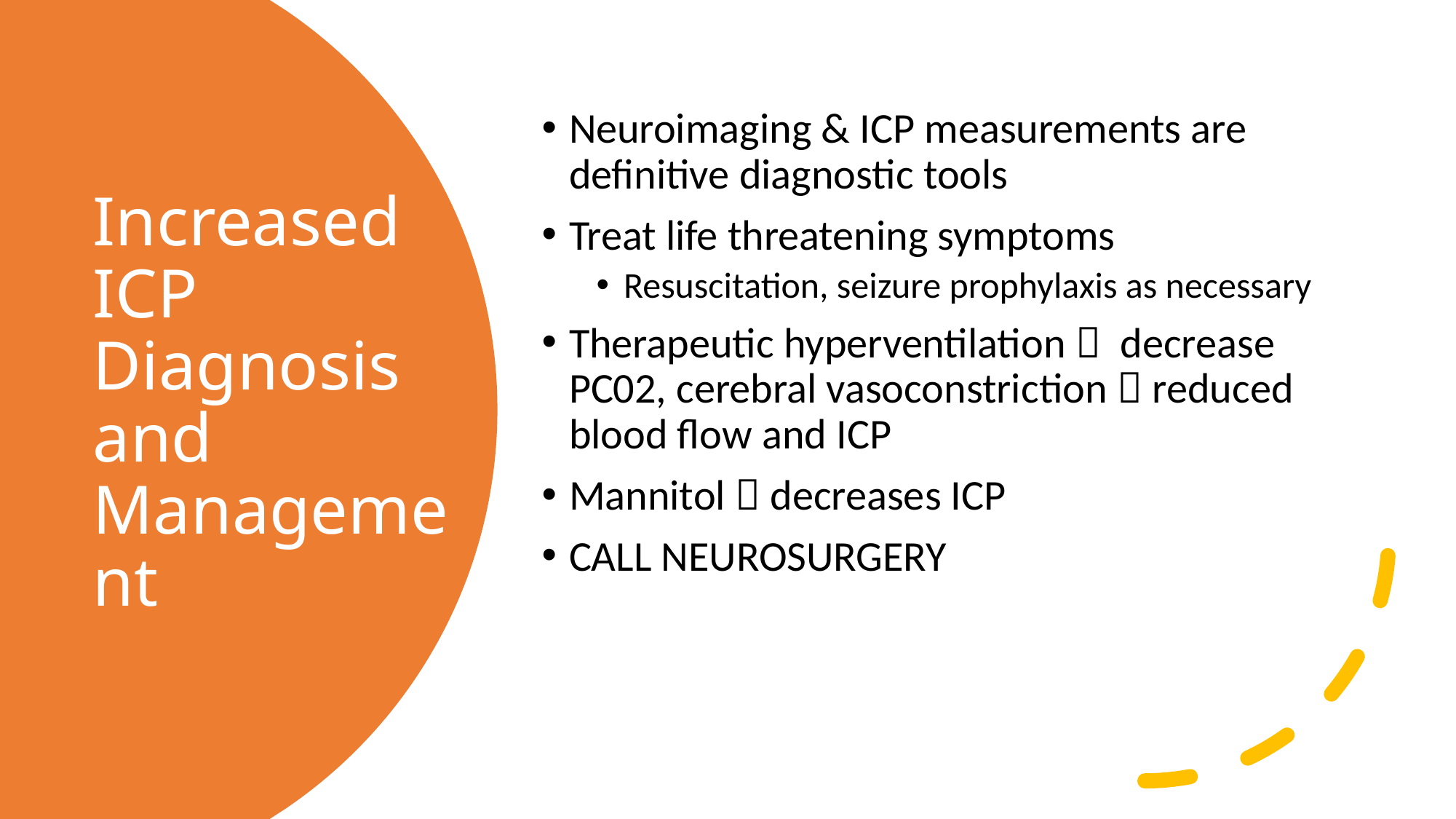

Neuroimaging & ICP measurements are definitive diagnostic tools
Treat life threatening symptoms
Resuscitation, seizure prophylaxis as necessary
Therapeutic hyperventilation  decrease PC02, cerebral vasoconstriction  reduced blood flow and ICP
Mannitol  decreases ICP
CALL NEUROSURGERY
# Increased ICP Diagnosis and Management

## Slide 8
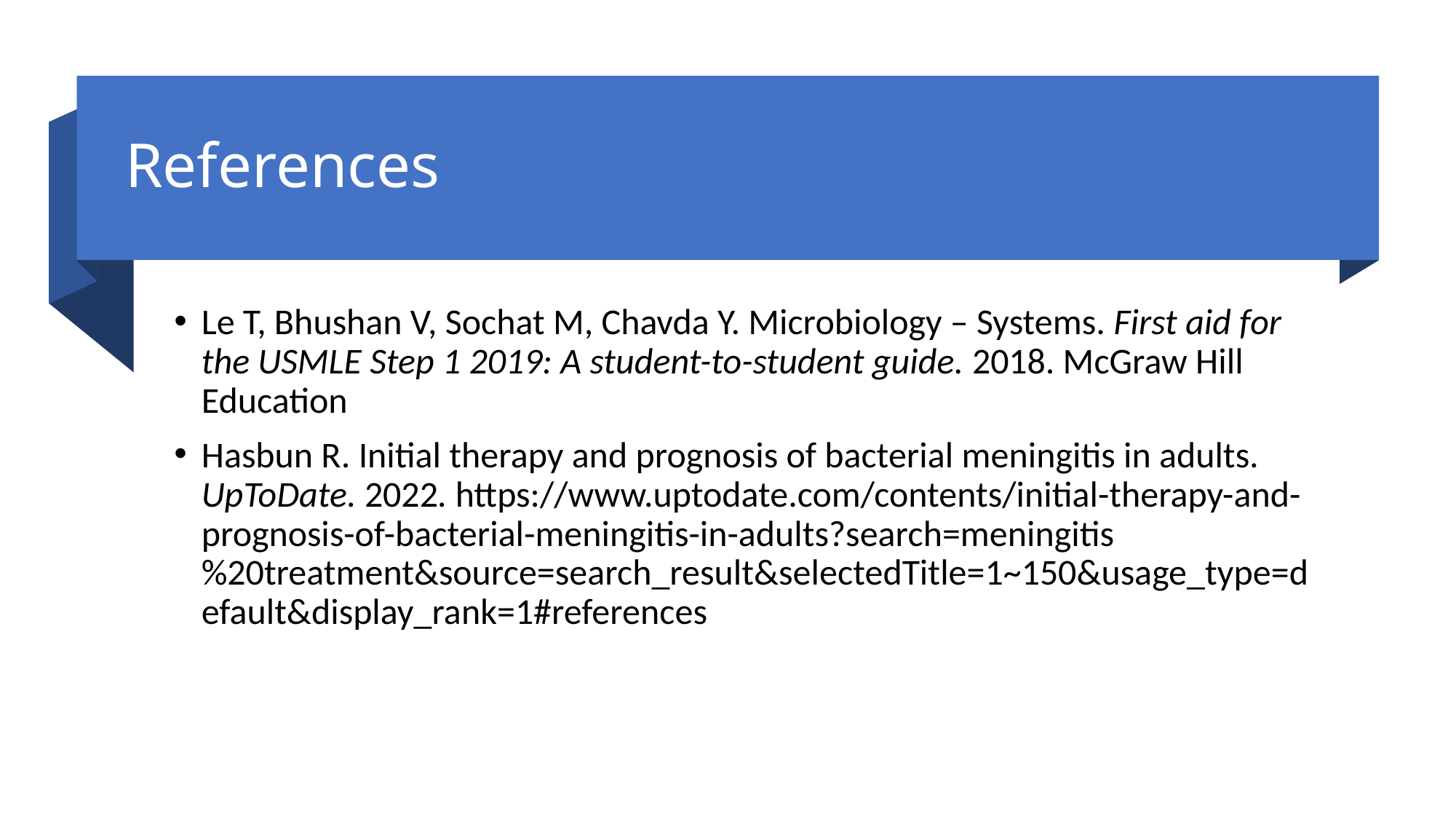

# References
Le T, Bhushan V, Sochat M, Chavda Y. Microbiology – Systems. First aid for the USMLE Step 1 2019: A student-to-student guide. 2018. McGraw Hill Education
Hasbun R. Initial therapy and prognosis of bacterial meningitis in adults. UpToDate. 2022. https://www.uptodate.com/contents/initial-therapy-and-prognosis-of-bacterial-meningitis-in-adults?search=meningitis%20treatment&source=search_result&selectedTitle=1~150&usage_type=default&display_rank=1#references
