# Supplementary material for: Bacterial Meningitis With Cerebral Edema in a Young Adult: A Simulation Case for Medical Students
Source: MedEdPORTAL. 2023 Oct 27;19:11354. doi: 10.15766/mep_2374-8265.11354 (PMC10603216; doi:10.15766/mep_2374-8265.11354)
Supplement: Supplementary file 1 — Simulation Case and Facilitator Guide.docxSimulation Images.docxLaboratory Values.docxPostencounter Questionnaire.docxMeningitis Debrief.pptx [file mep_2374-8265.11354-s001.zip › D. Postencounter Questionnaire.docx]

Appendix D: Post Encounter Questionnaire

The following questions were presented to the participants using an online survey platform and completed on their individual devices. All but the qualifying questions and free-response feedback were presented as a 5-point Likert scale. Questionnaire was adapted by the authors from the Simulation Effectiveness Tool – Modified.^1^

Qualifying Questions

- What is your year in medical school? (OMS-I, OMS-II, OMS-III, OMS-IV)
- Do you have any prior medical experience? (Scribe, EMT, Paramedic, Nurse, NP/PA, Pharmacist, Hospital volunteer, none, other)

Survey Questions

Using the Likert Scale (1 – strongly disagree, 2 – disagree, 3 – neutral, 4 – agree, 5 – strongly agree), please rate how you agree with the following statements.

| Survey Question | 1 | 2 | 3 | 4 | 5 | N/A |
| --- | --- | --- | --- | --- | --- | --- |
| 1. The simulation was a valuable learning experience. |  |  |  |  |  |  |
| 1. The simulation was realistic. |  |  |  |  |  |  |
| 1. The simulation was appropriate for my level of education/training. |  |  |  |  |  |  |
| 1. The simulation tested my clinical ability. |  |  |  |  |  |  |
| Please utilize this space to comment on any responses of 3 or less for questions 1-4. | | | | | | |
| The simulated case was effective at meeting the learning objectives of: |  |  |  |  |  |  |
| 1. Implement appropriate resuscitation measures for an acutely ill patient. |  |  |  |  |  |  |
| 1. Obtain appropriate history and physical exam to arrive at a differential diagnosis of meningitis. |  |  |  |  |  |  |
| 1. Analyze laboratory results to arrive at a differential diagnosis of meningitis. |  |  |  |  |  |  |
| 1. Analyze imaging results to arrive at a differential diagnosis of meningitis. |  |  |  |  |  |  |
| 1. Evaluate the need for empiric antibiotics in a potential neurologic infection. |  |  |  |  |  |  |
| 1. Evaluate the need for consultation with neurology and/or infectious disease. |  |  |  |  |  |  |
| 1. Perform procedural skill of lumbar puncture. |  |  |  |  |  |  |
| 1. Perform procedural skill of venous access. |  |  |  |  |  |  |
| 1. Perform procedural skill of endotracheal intubation. |  |  |  |  |  |  |
| Please utilize this space to comment on any responses of 3 or less for questions 5-13. | | | | | | |
| The skills portion of the event improved my comfort level of performing: |  |  |  |  |  |  |
| 1. Lumbar puncture |  |  |  |  |  |  |
| 1. Intravenous access |  |  |  |  |  |  |
| 1. Endotracheal intubation |  |  |  |  |  |  |
| Please utilize this space to comment on any responses of 3 or less for questions 14-16. | | | | | | |
| 1. The debrief was conducted in a safe environment |  |  |  |  |  |  |
| 1. The debrief elicited reflection and an opportunity for discussion |  |  |  |  |  |  |
| Please utilize this space to comment on the debrief session. | | | | | | |
| 1. The simulation event was an effective use of time. |  |  |  |  |  |  |
| 1. The allotted time for the simulated case was appropriate. |  |  |  |  |  |  |
| 1. The allotted time for the skills portion was appropriate. |  |  |  |  |  |  |
| Please utilize this space to comment on any responses of 3 or less for questions 19-21. | | | | | | |

Free Response

| 1. Please comment which portion of the event was most useful. |
| --- |
| 1. Please comment which portion of the event was least useful. |
| 1. Please describe how this simulation will impact your clinical abilities? |
| 1. Please provide any suggestions on how the simulated case could be improved. |
| 1. Please provide any suggestions on how the learning event could be improved. |
| 1. Please provide any other feedback. |

References

1. Leighton K, Ravert P, Mudra V, Macintosh C. Updating the simulation effectiveness tool: Item modification and reevaluation of psychometric properties. *Nursing Education Perspectives.* 2015;36(5):317-323. <https://doi.org/10.5480/15-1671>
