# Supplementary material for: Bacterial Meningitis With Cerebral Edema in a Young Adult: A Simulation Case for Medical Students
Source: MedEdPORTAL. 2023 Oct 27;19:11354. doi: 10.15766/mep_2374-8265.11354 (PMC10603216; doi:10.15766/mep_2374-8265.11354)
Supplement: Supplementary file 1 — Simulation Case and Facilitator Guide.docxSimulation Images.docxLaboratory Values.docxPostencounter Questionnaire.docxMeningitis Debrief.pptx [file mep_2374-8265.11354-s001.zip › A. Simulation Case and Facilitator Guide.docx]

| **Appendix A: Simulation Case and Facilitator Guide**  **SIMULATION CASE TITLE:** Bacterial Meningitis with Cerebral Edema in a Young Adult: A Simulation Case for Medical Students  AUTHORS: Kyle Cohen, Grant Gregory, Dianne Walker, James Nolin, MSN, Alexandra Sappington, Jonathan Hardy, Julia Alexander, MD, John Giannini, MD  **LEARNER AUDIENCE:** Pre-clinical medical students | | |
| --- | --- | --- |
| **PATIENT NAME:** Nicholas Payne  **PATIENT AGE:** 28 years old  **CHIEF COMPLAINT:** “He has been vomiting and confused”  **PHYSICAL SETTING:** Emergency Department | | |
| **Brief narrative description of case**  *Include the presenting patient chief complaint and overall learner goals for this case* | 28-year-old male presents to the ED with confusion and vomiting for last 2 days. Family states he was diagnosed with a viral URI a couple of weeks ago. Has complained of headache, facial pain, neck pain, and vomiting that has progressively worsened over the last couple of days. Lumbar puncture reveals bacterial meningitis and is correlated with head CT that also shows cerebral edema with early stages of herniation. |  |
| **Primary Learning Objectives**  *What should the learners gain in terms of knowledge and skill from this case? Use action verbs and utilize Bloom’s Taxonomy as a conceptual guide* | By the end of this activity, learners will be able to:   1. Implement appropriate resuscitation measures for an acutely ill patient. 2. Obtain appropriate history and physical exam to arrive at a differential diagnosis of meningitis. 3. Analyze laboratory results to arrive at a differential diagnosis of meningitis. 4. Analyze imaging results to arrive at a differential diagnosis of meningitis. 5. Evaluate the need for empiric antibiotics in a potential neurologic infection. 6. Evaluate the need for consultation with neurology and/or infectious disease. 7. Perform procedural skill of lumbar puncture. 8. Perform procedural skill of venous access. 9. Perform procedural skill of endotracheal intubation. |  |
| **Critical Actions**  *List which steps the participants should take to successfully manage the simulated patient. These should be listed as concrete actions that are distinct from the overall learning objectives of the case.* | Critical Actions:  -Check airway, breathing, and circulation  -Obtain and interpret vital signs  -Initiate primary resuscitation by obtaining/requesting IV access and giving fluid bolus  -Administer antipyretic  -Obtain pertinent history from spouse  -Obtain labs (CBC, BMP, Liver Function, Coagulation profile, Urinalysis)  -Obtain blood cultures  -Perform lumbar puncture  -Request Head CT  -Consider endotracheal intubation  -Request post intubation chest X-ray if done  -Administer broad spectrum antibiotics (not required to know specific drug)  -Consult neurology/infectious disease for the management of meningitis  -Admit to intensive care unit  -Work effectively as a team |  |
| **Learner Preparation or Prework**  *What information should the learners be given prior to initiation of the case?* | Background: Nicholas Payne is a 28-year-old male that presents to the ED with projectile vomiting and altered mental status. He presented to an outpatient clinic 7 days ago with right hemifacial pain. Labs and exam were normal at that time and patient was discharged with Dx of viral upper-respiratory infection. Pt family states vomiting worsening over last 2 days and decreased level of consciousness. Spouse is available for history by phone (or in the room with the patient). |  |

| Initial Presentation | | | |
| --- | --- | --- | --- |
| **Initial vital signs** | BP 98/48mmHg HR 110 bpm Resp 18/min SpO2 96% RA Temp. 103.4 F (orally)  Point of care glucose: 100mg/dL | | |
| **Overall Setting and Appearance**  *What do learners see when they first enter the room? What environment are the learners in? What is the appearance of the mannequin?* | Patient is lying on hospital bed, unresponsive, wearing street clothes. Patient is already on monitor. Patient family member (spouse) may be in control room available by phone, or in room with patient if desired. | | |
| **Standardized Participants (and their roles in the room at case start**)  *Who is present at the beginning and what is their role? Who may play them? Describe what they should say (i.e., their verbal scripts).* | Spouse: Available in control room or by phone to provide history and answer questions. May also accompany patient in room if desired. Played by a standardized patient.  Simulation Staff: changes vitals and operates simulator, provides imaging and labs at certain points in scenario, acts as consults as needed. Ideally outside the room but may be in the room depending on type of simulator. | | |
| **HPI**  *Please specify what info here and below must be asked vs. what is volunteered by patient or other participants.*  *Information in italics and quotes is provided by the spouse when asked.* | Freely given from spouse:  *“He has been vomiting and confused. He had a sinus infection last week and went to the urgent care and they said it was just viral. Didn’t give him anything. Yesterday he started getting confused and threw up. Today it’s a lot worse and he’s not making any sense.”*  More HPI if asked:  Why did he come to the hospital? “*He has been vomiting and confused.”*  When did it start? *“2 days ago.”*  Does anything make it worse? *“He did say bright light made his headache worse”*  Does anything make it better? “*Not really”*  Any other symptoms? *“He said his face hurt last week and he had a headache”*  -If asked for anything else? *“He said his neck hurt too”*  -Review of systems positive for: Vomiting, fever, headache, neck pain, fatigue. All else negative  Social History if asked:  Are you married? *“Yes”*  Who lives at home? *“Just me, I’m his spouse.”*  What does he do for work? *“He works in construction.”*  Does he use tobacco? *“No”*  Has he used tobacco in the past? *“No”*  Does he drink alcohol? *“Yes, beer.”*  -If asked: How much does he drink? *“3 or 4 cans of beer on weekends.”*  Does he use any recreational drugs? *“No”*  What is his diet? *“Normal diet”* | | |
| **Past Medical/Surgical** | **Medications** | **Allergies** | **Family History** |
| None | None | None | Father, 62, alive, HTN  Mother, 60, alive, healthy  Brother, 34, alive, healthy |
| **Physical Examination** | | | |
| **General** | Unresponsive with periodic vomiting | | |
| **HEENT** | Eyes closed. Right eye mydriasis. Normocephalic, atraumatic | | |
| **Neck** | Supple. Neck rigidity on passive movement (verbalize if done). Positive Brudzinski test (verbalize if done) | | |
| **Lungs** | Clear, no increased work of breathing | | |
| **Cardiovascular** | No murmurs, gallops, or rubs | | |
| **Abdomen** | Soft nontender | | |
| **Neurological** | Unresponsive, GCS 3 | | |
| **Skin** | Dry, warm. No rashes, jaundice, or mottling | | |
| **GU** | Normal male, no incontinence | | |
| **Psychiatric** | N/A | | |

Preparation for the simulation

Facilitator: The facilitator, or simulation faculty, should create a scenario within the high-fidelity simulator software. It has been found that if the stages of the case are preset, it is much easier to operate than when trying to input or change vital signs throughout the case. Additionally, with the software, timers can be added and trends in vital signs can be done to show a prolonged deterioration in condition if no actions are taken.

Standardized participant: One standardized participant will act as the spouse. It is important for the standardized participant to be familiar with the case and script well before the beginning of the case. If the standardized participant attempts to read the script during the scenario, it can cause delays in response and negate some of the realism that is trying to be achieved with the case.

**Case Flow Sheet**

N Payne

Initial State (N Payne)

- Unresponsive; GCS 3; Right mydriasis
- Periodic vomiting

ACTIONS:
*IV Access Bolus *Bloodwork *Blood cultures
*EKG *CXR: Pre-Intubation *Lumbar puncture

*CXR: Post-Intubation (if done) *Head CT

HR: 110

RR: 14

SpO2: 96

Temp: 103.4

- 8 min

IV Saline Bolus & Antipyretics

- Blood Pressure improves
- Fever decreases
- May also recognize increased ICP based on presentation and ventilate

HR: 100 BP: 108/60 RR: 14

SpO2: 96% Temp:101.2 F

No Bolus & Antipyretics

- Trends over 3 minutes to this set of vitals
- Should give fluids and antipyretics
- Should also ventilate

HR: 60 BP: 140/100 RR: 12

SpO2: 86% Temp: 103.8 F

Continued No Action = Herniation

BP: 150/120 Continued decompensation

HR: 30

RR: 8

SpO2: 80

Temp: 103.8

Head CT

- Head CT, provide read if requested
- If on track, give read that does NOT suggest meningitis
- If struggling, give read suggesting meningitis

HR: 98 BP: 112/68 RR:14

SpO2: 98% Temp: 100.2 F

Labs, LP, Antibiotics

- Provide lab results
- If LP requested, give results
- If intubated, provide post intubation CXR

HR: 90 BP: 110/66 RR: 18

SpO2: 98% Temp: 99.6 F

PEA - Idioventricular - *Scenario Over*

Participants may intubate at any time to manage the increased intracranial pressure. If they place patient on the ventilator, ask them what rate they want for ventilations. Appropriate answer is either rate of 18-20 or to measure of capnography.

Consultation

- Consult neuro &/or infectious disease

HR: 89 BP: 116/70 RR: 18

SpO2: 98% Temp: 99.6 F

They may also give mannitol after head CT suggests increased ICP.

**Ideal Scenario Flow**

After reading the “Background” in the learner preparation section, the facilitator announces, “Begin scenario.” They should assess the patient’s responsiveness to verbal and painful stimuli and recognizes a GCS 4 (E1V2M1). The patient is already placed on the monitor and the learners should recognize slight hypotension, tachycardia, and high fever. The learners should initiate IV access, IV fluid bolus, and antipyretics. The learners should assess for causes of AMS by looking at pupils and checking fingerstick glucose. When assessing pupils, right mydriasis is present and should direct the learners to obtain a head CT. There is a read of the CT that can be presented to the learners, or they can consult radiology and the facilitator can provide those read results verbally. There is also history available via phone from the patient’s spouse that should drive the learners toward a differential of meningitis. The patient had a “viral URI” for the past week and has increased headache, photophobia, neck pain, vomiting, and confusion. The learners should obtain a lumbar puncture and other labs including CBC, CMP, urinalysis, and coagulation profile. Other labs that may be obtained include ABG, toxicology screen, and CRP. After finding an elevated WBC count, the learners should also order blood cultures and administer broad spectrum antibiotics and steroids. At any point, the learners may choose to intubate the patient to secure the airway. Learners may also administer mannitol as a treatment for the increased intracranial pressure. After stabilizing the patient, obtaining the appropriate labs, and administering antibiotics, the learners should consult neurology and/or infectious disease for admission to the ICU.

**Anticipated Management Mistakes**

*Provide a list of management errors or difficulties that are commonly encountered when using this simulation case.*

1. Failure to assess pupils in patient with AMS: This was a common mistake noted in many participants. Failure to assess the pupils often resulted in the failure to recognize the need for a head CT and the ultimate failure to recognize increased intracranial pressure and cerebral edema. If this occurs, the spouse may indicate that the patient has been very confused and that he had been complaining of the light hurting his eyes. If learner’s fail to recognize this in a timely manner, or require prompting, it should be discussed in the debriefing.
2. Failure to recognize meningitis as differential diagnosis based on history: This was missed by a couple of groups as they did not elicit quality history information from the patient’s spouse. This resulted in the delayed management of patient however, they ultimately circled back and obtained the needed history.
3. Failure to recognize meningitis as a possible cause of the cerebral edema: While our learners were able to ultimately arrive at the diagnosis of meningitis, it is possible that learners may not put the pieces of the history, physical, and head CT together. If the learners are struggling at coming with a diagnosis, the facilitator may provide the radiology interpretation that suggests a clinical correlation with meningitis or other cause of cerebral edema.
4. Failure to recognize or respond to critical vital signs: While our learners were able to identify and respond to the critical vital signs appropriately, it is possible that students would get tunnel vision and not notice this. If this happens, the facilitator operating the simulator may use the intercom, or step in the room, to act as the nurse and bring the learner’s attention to the vital signs. If learner’s fail to recognize this in a timely manner, ore require prompting, it should be discussed in the debriefing.
5. Failure to consult neurology/infectious disease or admit to the ICU: While our learners were all able to consult and ultimately admit the patient, it is possible that learners may be hung up here and not know how to provide a disposition for the patient. If this happens, the facilitator operating the simulator may use the intercom, or step in the room, to act as the nurse and make a comment such as “this patient seems really sick, should we consult a specialist?” or “Are we going to keep this patient in the ED all day?” If learner’s fail to recognize this in a timely manner, ore require prompting, it should be discussed in the debriefing.

Pertinent Findings

Physical exam:

Head CT: The head CT provided in Appendix B reflect cerebral edema and the clinical picture at the initiation of the case reflects increased intracranial pressure. There are two radiology interpretations for the CT, one of which hints toward meningitis, while the other does not. If the learners are struggling, the facilitator may provide the learners with the radiology interpretation that suggests a clinical correlation with meningitis.

Lab Values: The results of the BMP reflect a clinical picture of dehydration with a BUN of 50 and Creatinine of 2.5. The urinalysis correlates with dehydration as well with dark urine and an elevated specific gravity. The CBC suggests prolonged infection with a WBC count of 15,000 as well as slight anemia with a hemoglobin of 12.8 and hematocrit of 35.

Lumbar puncture analysis: This correlates bacterial meningitis with increased WBCs, protein, neutrophils, and CSF lactate, and a decreased glucose. These are important findings for the distinction between bacterial, viral/aseptic, and fungal meningitis. The increased RBCs is the result of a traumatic LP procedure.

Toxicology screen: A toxicology screen is available and may be read verbally to the learners should they ask for it. It is possible for the learners to request this as part of their work up for an unresponsive adult, but the findings are negative.

C-reactive protein (CRP): Should the learners request a CRP; it can be given to them verbally. The value will be elevated. CRP has traditionally been a marker that is found to be elevated in bacterial meningitis.

Serum lactate: Should the learners request a serum lactate; it can be given to them verbally. The value is slightly elevated reflecting possible sepsis, dehydration, prolonged vomiting, or other causes of tissue metabolism disturbance.

**Simulation Environment Checklist**

This checklist may be utilized prior to the simulation to ensure that the high-fidelity environment is adequately prepared for the learners. The environment and resources should reflect that of what is typically available in the learners’ practice environment. For this particular event, all interventions beyond simple airway management and oxygenation were verbalized and skills were practiced on low fidelity simulators. The equipment that we utilized is provided in a checklist below. However, if time permits and resources are available, all skills could be performed and evaluated as part of the overall simulated patient encounter.

**Equipment**

- Adult High-fidelity simulator with operator computer
- Patient monitor, ideally connected to high-fidelity simulator
- Speaker system for communication with SP acting as the spouse (the spouse may also be available in the room if no speaker system is available)
  - If in the room, a chair should be provided.
- Hospital bed
- Simulated Suction with Yankauer tip
- AED/Defibrillator with pads or paddles
- Airway Management supplies
  - Non-rebreather mask
  - Simple face mask
  - Oral airways sized 8-11
  - Nasal airways sized 6-9
  - Laryngoscope handle
  - Macintosh blades 2, 3, 4
  - Miller blades 2, 3, 4
  - Cuffed endotracheal tubes (ETT) sizes 6.0, 6.5, 7.0, 7.5
  - 10cc syringe
  - Adult stylet
  - Gum bougie
  - End-tidal capnography adapter
  - McGill forceps
  - ETT securing device or tape
- IV supplies
  - Tourniquet
  - Alcohol prep pads
  - IV catheters various sizes
  - Saline lock tubing or similar
  - Syringes
  - Saline flushes
  - 4x4 gauze pads
  - Tape
  - Tegaderm
  - Specimen collection tubes
- Lumbar puncture tray

**Props**

- Ball cap for high-fidelity simulator
- Street clothes for high-fidelity simulator

**Medications** (in our event, medications were verbalized. This is a list of anticipated medications should you chose to have the learners administer them during the scenario)

- IV fluids (Normal saline or Lactated Ringers)
- Acetaminophen
- Adenosine
- Amiodarone
- Ampicillin
- Atropine
- Calcium chloride
- Cephalosporin (Ceftriaxone or cefotaxime)
- Dexamethasone
- Dextrose 50%
- Diazepam
- Dopamine
- Epinephrine 1:10,000
- Epinephrine 1:1,000
- Etomidate
- Fentanyl
- Flumazenil
- Glucagon
- Hydrocortisone
- Ibuprofen
- Ketamine
- Lidocaine
- Lorazepam
- Mannitol
- Midazolam
- Morphine
- Naloxone
- Piperacillin/Tazobactam
- Prednisolone
- Rocuronium
- Sodium bicarbonate
- Succinylcholine
- Vancomycin
- Vecuronium

Task Trainers

- Intravenous access arm
  - IV Supplies as listed above
- Endotracheal intubation/airway management head
  - Airway management supplies as listed above
- Lumbar puncture back
  - Lumbar puncture tray

Simulation Checklist

This checklist may be utilized by the facilitator to keep track of items that are ordered as well as critical actions of the case to evaluate the learners and provide feedback during the debrief session. As space for notes is provided below each action.

Critical Actions

- Check airway, breathing, and circulation
- Obtain/request and interpret vital signs
- Initiate primary resuscitation by obtaining/requesting IV access and giving fluid bolus
- Administer/request antipyretic
- Obtain pertinent history from spouse
- Obtain/request labs (CBC, BMP, Liver Function, Coagulation profile, Urinalysis)
- Obtain/request blood cultures
- Perform/request lumbar puncture with analysis of fluid
- Request Head CT
- Consider endotracheal intubation
  - Request post intubation chest X-ray if intubated
- Administer/request broad spectrum antibiotics (not required to know specific drug)
- Consult neurology
- Admit to intensive care unit
- Work effectively as a team

Detailed Schedule of Simulation Event

| Timeline | *Pre-Brief* | *Simulation Room 1* | *Simulation Room 2* | *Skills Practice* | *Debrief* |
| --- | --- | --- | --- | --- | --- |
| Start | ABCD |  |  |  |  |
| Personnel | 2 facilitators |  |  |  |  |
| 5 Minutes |  | A | B | CD |  |
| Personnel |  | 1 SP, 1 facilitator | 1 SP, 1 facilitator | 3 facilitators/faculty |  |
| 25 Minutes | Reset and rotate | | | | |
| 30 Minutes |  | C | D | AB |  |
| Personnel |  | 1 SP, 1 facilitator | 1 SP, 1 facilitator | 2 facilitators/faculty |  |
| 50 Minutes |  |  |  |  | ABCD |
| Personnel |  |  |  |  | 2 facilitators |
| 60 Minutes | Wrap up and survey | | | | |

The letters “ABCD” denote the groups of learners and the appropriate rotation.
